# POU2F2 as a dual oncogenic and immunomodulatory driver in kidney renal clear cell carcinoma

**DOI:** 10.1080/19336918.2026.2700062

**Published:** 2026-07-08

**Authors:** Gonglin Tang, Fei Liu, Mingze Sun, Chenyue Liu, Jitao Wu, Hongwei Zhao

**Affiliations:** a Department of Urology, The Affiliated Yantai Yuhuangding Hospital of Qingdao University, Yantai, Shandong, China; b School of Clinical Medicine, Shandong Second Medical University, Weifang, China

**Keywords:** Immune infiltration, kidney renal clear cell carcinoma, macrophage, POU2F2, prognostic biomarker

## Abstract

Kidney renal clear cell carcinoma (KIRC) features an immunosuppressive tumor microenvironment (TME). We investigated the role of transcription factor POU2F2 in KIRC oncogenesis and macrophage polarization. Single-cell and spatial transcriptomics showed that POU2F2 is upregulated in KIRC and associated with metastasis and poor prognosis. POU2F2 knockdown inhibited KIRC cell proliferation, migration, and invasion in vitro. POU2F2 expression correlated with macrophage infiltration and JAK-STAT signaling. Co-culture assays showed that POU2F2 silencing reduced macrophage recruitment and shifted M2 polarization toward an anti-tumor M1 phenotype. Overall, POU2F2 acts as an oncogenic driver and prognostic biomarker in KIRC, promoting an immunosuppressive TME and representing a potential therapeutic target.

## Introduction

Kidney renal clear cell carcinoma (KIRC) represents the most prevalent and aggressive subtype of kidney cancer, accounting for approximately 75% of renal malignancies [1]. Despite advancements in molecular targeted therapies and immunotherapy, the prognosis for metastatic KIRC remains dismal due to intrinsic resistance mechanisms and tumor heterogeneity [2]. The immunosuppressive tumor microenvironment (TME), characterized by dysfunctional immune cell infiltration and aberrant intercellular signaling, further complicates therapeutic outcomes [2]. Recent studies have emphasized the critical role of transcriptional regulators in shaping the TME and driving KIRC progression. Among these, the POU-domain transcription factor family, particularly POU2F2 (also known as OCT2), has emerged as a multifaceted player in cancer biology, though its role in KIRC remains underexplored [3,4].

POU2F2, a member of the POU-domain transcription factor family, is indispensable for B-cell development and immunoglobulin gene expression. Its oncogenic potential has been highlighted across multiple malignancies. For instance, in glioblastoma (GBM), POU2F2 overexpression correlates with poor survival, where it modulates glycolytic reprogramming via the PDPK1-dependent PI3K/AKT/mTOR pathway, fueling tumor proliferation and immune evasion [5]. Conversely, in non-small cell lung cancer (NSCLC), POU2F2+ B cells exhibit antitumor properties by enhancing CD8+ T cell infiltration and M1 macrophage polarization, suggesting context-dependent roles in cancer immunity [6]. This duality underscores the need to delineate POU2F2’s functions within KIRC-specific contexts, where its interactions with stromal and immune components may differ substantially.

In KIRC, epithelial-to-mesenchymal transition (EMT) and metabolic reprogramming are hallmark features driving metastasis and therapeutic resistance. Single-cell transcriptomic analyses have identified aberrantly activated intercellular signaling pathways in KIRC, including those mediated by cancer stem cell (CSC) subpopulations expressing markers like POU5F1 and CD44 [2]. Intriguingly, POU2F2 has been implicated in regulating EMT-related transcription factors such as ZEB2, a key mediator of metastasis in multiple cancers [3]. In monocytes, POU2F2 co-expression with ZEB2 suggests a potential role in modulating immune cell plasticity within the TME, though this interplay remains uncharacterized in KIRC [3]. Furthermore, SETD2 loss—a frequent genetic alteration in KIRC—triggers EMT independently of TGF-β, hinting at alternative regulatory axes where POU2F2 might participate [7]. These findings collectively position POU2F2 as a plausible orchestrator of both CSC maintenance and EMT in KIRC. The immunomodulatory functions of POU2F2 further warrant investigation in KIRC.

In NSCLC, POU2F2+ B cells recruit antitumor immune populations, whereas in GBM, POU2F2 promotes an immunosuppressive niche by upregulating PD-L1 and inhibiting NK cell activity [5,6]. Such opposing effects highlight tissue-specific regulatory networks. In KIRC, where immune checkpoint inhibitors (ICIs) show limited efficacy, understanding how POU2F2 influences immune infiltration—particularly myeloid-derived suppressor cells (MDSCs) and tumor-associated macrophages (TAMs)—could unveil novel combinatorial strategies. For example, POU2F2’s interaction with SWI/SNF chromatin remodelers, as observed in small-cell lung cancer (SCLC), may modulate enhancer regions governing immune checkpoint molecule expression. Additionally, its co-activator role for POU2F3 in SCLC underscores its potential to regulate lineage-specific transcriptional programs in KIRC [8]. In breast cancer, POU2F2 intersects with pathways governing iron and copper homeostasis, suggesting its broader role in metabolic adaptation. Whether similar mechanisms operate in KIRC remains unknown but merits exploration [9]. Despite these insights, critical gaps persist. First, the prognostic significance of POU2F2 in KIRC has not been systematically evaluated. Second, its regulatory crosstalk with key KIRC drivers—such as VHL mutations, HIF-2α, or immune checkpoints—remains elusive. Third, the therapeutic potential of targeting POU2F2 or its downstream effector molecules in KIRC has not been explored. This study aims to interrogate the roles of POU2F2 in KIRC progression, immune evasion, and related oncogenic processes by integrating multi-omics datasets, functional assays, and preclinical models. Our findings may furnish both theoretical and experimental foundations for therapeutic strategies that exploit POU2F2 as a dual oncogenic driver and immunomodulatory factor in this intractable malignancy.

## Materials and methods

### Data acquisition

The Cancer Genome Atlas (TCGA), accessed via UCSC Xena (https://genome-cancer.ucsc.edu/), constitutes an expansive, multi-omics compendium of high-depth molecular profiles spanning 33 histologically discrete cancer types—including KIRC. The resource integrates whole-exome and whole-genome sequences, transcriptomes, methylomes, quantitative proteomes, and longitudinal clinical annotations. From this public repository we extracted POU2F2 expression quantifications, survival outcomes, and exhaustive clinicopathological covariates for the TCGA-KIRC cohort.

### The gene expression omnibus and human protein Atlas databases

The Gene Expression Omnibus (GEO; https://www.ncbi.nlm.nih.gov/geo/) is a MIAME-compliant, community-driven archive that curates and disseminates high-throughput microarray, RNA-seq, and other functional genomic datasets, empowering integrative querying, downstream analysis, and interactive visualization [10]. The Human Protein Atlas (HPA; https://www.proteinatlas.org) is an expansive proteomic cartography initiative that systematically delineates the spatiotemporal expression landscape of virtually every human protein across cells, tissues, and organs. Leveraging rigorously validated immunohistochemical micrographs, the atlas furnishes quantitative expression maps, subcellular localization patterns, functional annotations, and disease-association metrics for the majority of the human proteome.

### Univariable and multivariable logistic regression modeling

Univariate Cox modeling quantified the association between POU2F2 transcript abundance and overall survival in two independent KIRC cohorts, delineating its prognostic relevance. Multivariable Cox regression—adjusting for established clinicopathological confounders—was then implemented to evaluate POU2F2 as an autonomous survival determinant. Prognostic nomograms and calibration plots were subsequently generated with the rms package to provide individualized risk estimation and internal validation.

### Dissection of oncogenic cascades and mechanistic underpinnings

To dissect POU2F2-centered signaling networks and their mechanistic underpinnings, we dichotomized pan-cancer specimens into high- and low-expression cohorts at the 50th percentile of POU2F2 abundance and interrogated differential transcriptomes between strata. Interactors of POU2F2 were next retrieved from ComPPI (https://comppi.linkgroup.hu/), a compartment-resolved interactome that consolidates multi-source PPI evidence with subcellular localization metrics, thereby refining the molecular neighborhood of POU2F2. Functional annotation proceeded through gene-set enrichment (GSEA) and KEGG pathway profiling to delineate the oncogenic cascades and metabolic circuits in which POU2F2 is embedded [11]. We further quantified perturbations across 50 hallmark signatures and 91 metabolic modules in POU2F2-high versus POU2F2-low tumors and evaluated Pearson correlations between POU2F2 abundance and 14 predefined biological processes, thereby illuminating the multifaceted role of POU2F2 in renal carcinogenesis.

### Leveraging the TISCH2 compendium to dissect POU2F2-mediated immune reprogramming within the renal carcinoma microenvironment

Tumor Immune Single-cell Hub 2 (TISCH2; http://tisch.comp-genomics.org/) curates an expansive compendium of single-cell RNA-sequencing profiles interrogating the TME. Spanning 190 datasets across 50 malignancies and encompassing >6.3 million high-resolution cellular transcriptomes, this atlas delivers granular cell-type taxonomies that empower pan-cancer dissection of TME heterogeneity. We mined the repository to extract KIRC-relevant scRNA-seq cohorts and chart POU2F2 expression dynamics within the renal TME.

### Tumor-immune system interaction database

Tumor-Immune System Interaction Database (http://cis.hku.hk/TISIDB) is a comprehensive integrative portal that amalgamates multi-omic and clinical datasets to decode tumor–immune crosstalk, delineating how gene-centric perturbations sculpt anti-tumor immunity. Incorporating immunotherapy-treated patient cohorts alongside functional genomics screens and TCGA pan-cancer resources, the repository enables quantitative appraisal of immunotherapeutic responsiveness, dissection of neoplastic–immune cell interplay, and correlation analyses linking intratumoral lymphocytic infiltrates to gene expression landscapes.

### Immune infiltration analysis

GSVA (Gene Set Variation Analysis)-mediated single-sample gene set enrichment analysis (ssGSEA) was deployed to quantify the abundance of 24 immune cell subsets; subsequent comparisons between POU2F2-high and POU2F2-low tumors employed Spearman correlation and Wilcoxon rank-sum tests to gauge the magnitude of association. All graphical outputs were rendered with ggplot2.

### Cell purchase and culture

Human KIRC lines 786-O, 769-P, ACHN, Caki-2, and A498, alongside the non-neoplastic proximal tubular epithelial line HK-2, were procured from the Cell Bank of the Chinese Academy of Sciences. KIRC cells were propagated in RPMI-1640 and HK-2 in DMEM (BI, Israel), each supplemented with 10% (v/v) fetal bovine serum. Cultures were sustained at 37°C under a humidified 5% CO_2_ atmosphere.

### Isolation of total RNA, complementary DNA synthesis, and quantitative reverse-transcription PCR

Total RNA was isolated from KIRC cell lines using the RNeasy Kit (Qiagen, China) and reverse-transcribed with Evo M-MLV RT Master Mix (Accurate Biology, China). Quantitative PCR was subsequently performed on a 7500 Real-Time PCR System (Applied Biosystems) using SYBR Green Premix Pro Taq HS qPCR Kit plus ROX passive reference (Accurate Biology, China). GAPDH served as the endogenous control, and relative gene expression was calculated by the 2–ΔΔCT method; all reactions were run in triplicate [12]. Each experiment was conducted in triplicate, and the primer sequences used are presented in Table 1.

**Table 1.** Sequence of gene-specific primers for qPCR.

Gene	Forward sequence (5’− 3’)	Reverse sequence (5’− 3’)
POU2F2	TCCTGGAGAAGTGGCTCAACGA	ATGCTGGTCCTCTTCTTGCGTC
GAPDH	GTCTCCTCTGACTTCAACAGCG	ACCACCCTGTTGCTGTAGCCAA
CD86	CCATCAGCTTGTCTGTTTCATTCC	GCTGTAATCCAAGGAATGTGGTC
CD206	AGCCAACACCAGCTCCTCAAGA	CAAAACGCTCGCGCATTGTCCA
IL10	TCTCCGAGATGCCTTCAGCAGA	TCAGACAAGGCTTGGCAACCCA
iNOS	GCTCTACACCTCCAATGTGACC	CTGCCGAGATTTGAGCCTCATG
IL6	AGACAGCCACTCACCTCTTCAG	TTCTGCCAGTGCCTCTTTGCTG
CSF-1	TGAGACACCTCTCCAGTTGCTG	GCAATCAGGCTTGGTCACCACA
ARG1	TCATCTGGGTGGATGCTCACAC	GAGAATCCTGGCACATCGGGAA

### Transfection

POU2F2-targeting shRNAs and negative-control lentiviral vectors were custom generated by OBiO Technology (China) and transduced into KIRC cells using polybrene-assisted infection according to the manufacturer’s instructions, the target sequence is shown in Supplementary Table S1. Knockdown efficiency was subsequently validated by qRT–PCR and Western blot.

### CCK-8 assay

ACHN and 769-P cells stably expressing either NC or shPOU2F2 were seeded into 96-well plates (2 × 10^3^ cells/well), and viability was serially monitored at 0, 24, 48, 72, and 96 h by CCK-8 assay (CCK-8; Dojindo, Japan); absorbance at 450 nm was quantified after 2 h incubation at 37°C.

### Wound healing assay

Cells were plated at 2 × 10^6^ per well in 6-well plates, allowed to adhere overnight, and wounded with a sterile 1000-µl pipette tip to generate uniform scratches. Following PBS rinsing, baseline images were acquired microscopically (0 h). Wells were replenished with 2 mL RPMI-1640 complete medium, and identical fields were re-imaged after 12 h; residual wound area served as a quantitative read-out of migratory capacity [13].

### Transwell invasion experiment

6 × 10^4^ cells were plated atop Matrigel-coated Transwell inserts and allowed to invade toward a 10% FBS chemoattractant for 24 h; thereafter, penetrated cells were paraformaldehyde-fixed, crystal-violet-stained, and enumerated by bright-field microscopy.

### Western blot

The cells were lysed in RIPA buffer (Servicebio, China) containing 1% protease inhibitor for 30 minutes, followed by further sonication on ice using a sonicator at approximately 15% power for 10–15 seconds. The resulting protein supernatant was obtained after centrifugation. The supernatant was then mixed with 5× protein loading buffer (Coolaber, China) and heated at 100°C for 10 minutes for sample preparation. SDS-PAGE was performed for electrophoresis, and the proteins were transferred onto a PVDF membrane (Millipore, USA). The membrane was blocked with TBST buffer containing 5% skim milk for 2 hours at room temperature, and then incubated with various primary antibodies overnight at 4°C on a shaker. The membrane was subsequently incubated with secondary antibodies at room temperature for 1 hour. The ECL substrate solution A and B were mixed in equal proportions to prepare the developing solution, which was evenly dropped onto the PVDF membrane and visualized using a protein imaging system. The antibodies used were as follows: rabbit polyclonal POU2F2 antibody (Proteintech, China, 1:1000), rabbit monoclonal β-actin antibody (Proteintech, China, 1:1000) and HRP-conjugated goat anti-rabbit secondary antibody (Proteintech, China, 1:5000).

### Co-culture experiments of KIRC cells and macrophages

The human macrophage cell line THP-1 was maintained in RPMI-1640 medium and stimulated with 100 nM phorbol 12-myristate 13-acetate (PMA) for 24–48 h to induce macrophage differentiation, with concomitant supplementation of 25 nM macrophage colony-stimulating factor (M-CSF). After 24 h of PMA treatment, macrophages (5 × 10^4^ cells/mL) were seeded into the upper chamber in 200 μL RPMI-1640 containing 10% FBS. Control and knockdown KIRC cells were plated in the lower chamber at 5 × 10^4^ cells/mL in 500 μL medium containing 10% FBS. Migrated macrophages in the lower chamber were stained with crystal violet, and a Transwell assay was performed to evaluate changes in macrophage migratory capacity following co-culture with KIRC cells. Subsequent macrophage assays were conducted after co-culture with conditioned media from ACHN or 769-P cells with or without POU2F2 knockdown. All co-culture experiments were performed using a 24-well Transwell system (8.0 μm pore size; Cat. No. 3342; Corning, USA).

### Flow cytometry

To determine macrophage polarization, flow cytometry was performed. After co-culture, adherent macrophages were gently harvested using 0.25% trypsin-EDTA solution and were subsequently washed twice with PBS. The cells were then stained immediately without fixation, as live-cell analysis was performed. Macrophages were blocked with 10% FBS and incubated with antigen-specific fluorophore-conjugated antibodies (CD86: 374,216, BioLegend, USA; CD206: 321,106, BioLegend, USA) at 4°C in the dark for 2 h. Cells were washed twice with 3 mL flow cytometry buffer and resuspended in 400 μL flow cytometry buffer. A minimum of 50,000 events was acquired per sample on the FACSCalibur flow cytometer (BD Biosciences, USA), and data were analyzed using FlowJo v10.8 (FlowJo, USA).

### Statistical analysis

Analytical workflows were executed in R v4.2.1 and web-based platforms. Two-group comparisons of non-parametric variables were performed with the Wilcoxon rank-sum test; multi-group differences were evaluated via the Kruskal–Wallis test. Pearson correlations were applied to normally distributed data, whereas Spearman correlations served non-Gaussian distributions. Parametric contrasts employed Student’s t-tests or one-way Analysis of Variance (ANOVA) as appropriate. Statistical significance was defined at *p* < .05.

## Results

### Up regulation of POU2F2 expression in KIRC

**Figure 1.**
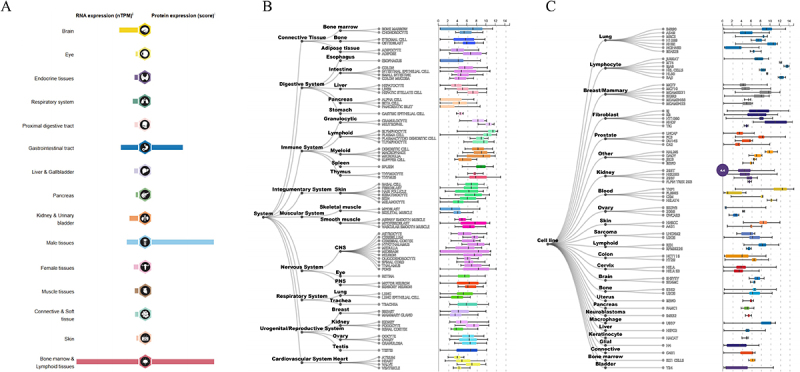
Overall expression profiles of POU2F2 at the mRNA and protein levels across human organs. (A) Overview of POU2F2 expression at the mRNA and protein levels across human organs. (B) Expression of POU2F2 across human systems, organs, and cell types. (C) Expression profile of POU2F2 across cell lines.

First, we systematically characterized the tissue- and system-wide distribution and expression profiles of POU2F2 at both the mRNA and protein levels across human organs using The Human Protein Atlas and Harmonizome 3.0, and further assessed its expression patterns across organ-derived cell lines (Figure 1(A–C)).

**Figure 2.**
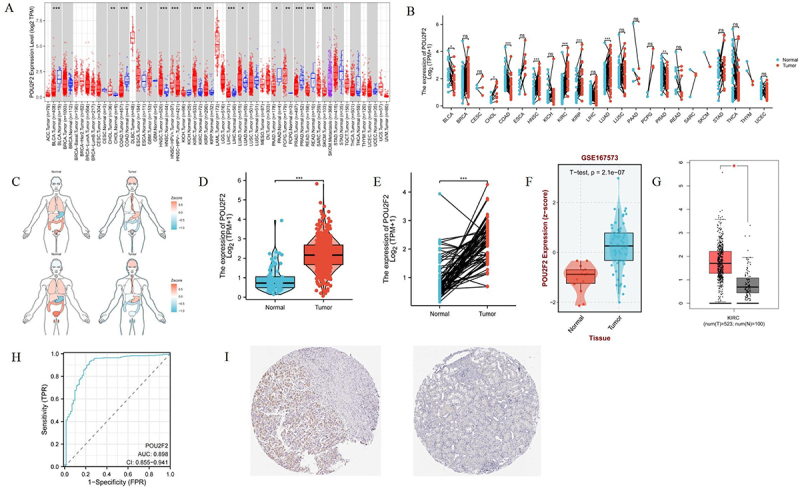
Pan-cancer and KIRC expression landscape of POU2F2. (A) the expression levels of POU2F2 across various tumors and normal tissues. (B) the expression profile of POU2F2 within paired tumor samples. (C) the expression and distribution of POU2F2 across tumor tissues from various organs and their corresponding normal tissues. (D) the expression levels of POU2F2 in KIRC tumor tissues compared to normal tissues. (E) the expression levels of POU2F2 in KIRC tumor tissues and their corresponding paired normal tissues. (F) POU2F2 expression levels in KIRC as documented in the GSE167573 dataset. (G) the expression levels of POU2F2 in KIRC, as documented in the GEPIA database. (H) Diagnostic potential of POU2F2 in KIRC evaluated by ROC curve analysis. (I) the HPA database reveals the expression levels of POU2F2 in KIRC tissues and normal tissues. (*p < .05, **p < .01, and ***p < .001).

To delineate the pan-cancer expression landscape of POU2F2, we interrogated TCGA datasets and generated a boxplot profiling its abundance across 33 malignancies (Figure 2(A)). POU2F2 was markedly overexpressed in 14 cancer types, including KIRC. Analysis of matched tumor–adjacent normal pairs revealed pronounced POU2F2 up-regulation in KIRC (Figure 2(B)). Stratification by sex further unveiled divergent POU2F2 expression patterns across organs, with consistent and significant elevations in malignant tissues (Figure 2(C)). Integrative interrogation of the TCGA-KIRC cohort revealed marked transcriptional up-regulation of POU2F2 in neoplastic versus adjacent normal tissue (Figure 2(D)); concordant observations were obtained from paired tumor–normal specimens (Figure 2(E)). Independent corroboration via POU2F2 and the GEO datasets GSE167573 substantiated the robust elevation of POU2F2 transcripts in KIRC (Figure 2(F,G)). The ROC curve analysis of POU2F2 showed that the area under the curve was 0.898 (Figure 2(H)) Immunohistochemical profiling within the Human Protein Atlas further demonstrated accentuated POU2F2 protein abundance and intensified immunoreactivity in malignant tissues relative to peri-tumoral counterparts (Figure 2(I)).

### POU2F2 transcriptional abundance in KIRC clinicopathologic associations and prognostic implications

**Figure 3.**
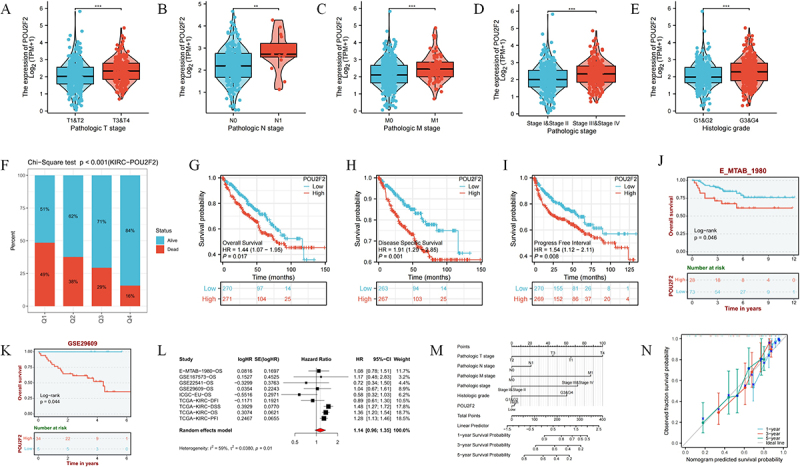
The relationship between POU2F2 expression and clinicopathological characteristics. (A-E) the correlation between POU2F2 and T, N, M stage, pathological stage and histologic grade in the TCGA database. (F) Chi-square tests were performed for the number of surviving and dead samples from subgroups with different POU2F2 expression levels. (G-I) the overall survival (OS), disease specific survival (DSS) and progression-free interval (PFI) survival curves for patients with high and low POU2F2 expression in KIRC. (J, K) OS survival curves of high and low POU2F2 expression in KIRC patients in data sets E_MTAB_1980 and GSE29609. (L) the meta-survival analysis for POU2F2. (M) a nomogram for predicting probability of patients with 1, 3 and 5-years overall survival. (N) the calibration curves of 1, 3 and 5-years survival of KIRC patients. (*p < .05, **p < .01, and ***p < .001).

Leveraging TCGA-KIRC transcriptomes, we interrogated the association between POU2F2 abundance and clinicopathologic variables. POU2F2 exhibited stepwise escalation across advancing T category (*p* < .001), nodal involvement (*p* < .001), distant metastasis (*p* < .001), higher pathological stage (*p* < .001), and escalating histologic grade (*p* < .001) (Figures 3(A–E)), implicating its expression as a surrogate of aggressive clear-cell renal carcinoma. Stratification into quartiles further revealed that the highest quartile (Q1) conferred the greatest mortality hazard (Figure 3(F)). Kaplan–Meier survival analysis of the TCGA-KIRC cohort revealed that elevated POU2F2 expression portended markedly diminished overall and progression-free survival (Figure 3(G–I)). External validation in the E-MTAB-1980 and GSE29609 cohorts corroborated that elevated POU2F2 transcription robustly predicts inferior survival in patients with KIRC (Figure 3(J,K)). A comprehensive meta-analysis of multiple KIRC cohorts confirmed that POU2F2 consistently conferred a hazard ratio >1, underscoring its designation as a high-risk prognosticator (Figure 3(L)). To translate these findings into individualized risk estimates, we constructed a nomogram integrating T stage, N stage, M stage, pathological grade, histologic grade, and POU2F2 abundance into a unified point scale that predicts 1, 3, and 5-year survival probabilities (Figure 3(M)). Calibration plots demonstrated excellent concordance between predicted and observed outcomes, validating the model’s accuracy (Figure 3(N)).

### Link between POU2F2 expression and pathways

**Figure 4.**
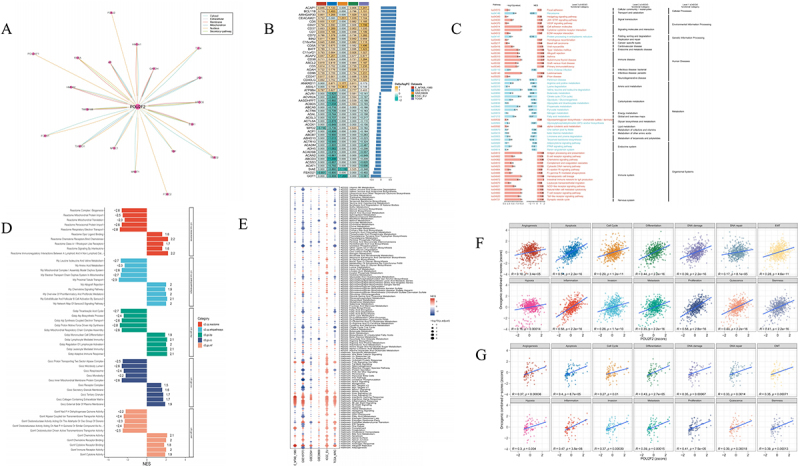
Analysis of pathways and underlying mechanisms. (A) PPI network of genes interacting with POU2F2 constructed from the ComPPI database. (B) Heatmap of differential genes in multiple cohorts of high and low POU2F2 expression groups. (C, D) KEGG and GSEA enrichment analysis results. (E) Enrichment analysis of various biological processes and pathways associated with POU2F2 in different databases. (F, G) Correlations between POU2F2 mRNA expression and fourteen oncogenic hallmarks in TCGA and ICGC datasets. (*p < .05, **p < .01, and ***p < .001).

The PPI network crafted through the ComPPI database offers a visual representation of genes interacting with POU2F2, including TMEM108, ESR1, XRCC6, and others (Figure 4(A)). To explore the role and mechanisms of POU2F2 in KIRC, we conducted differential analyses across numerous datasets and obtained robust results. We identified 25 genes that were significantly overexpressed and another 25 genes that were significantly underexpressed across multiple datasets. These consensus results suggest that POU2F2 is functionally related to or involved in the functions of these genes (Figure 4(B)). By conducting KEGG enrichment analysis on genes highly expressed in the high POU2F2 group, it is evident that POU2F2 expression is associated with JAK-STAT signaling pathway, Cytokine-cytokine receptor interaction, T cell receptor signaling pathway, and Natural killer cell mediated cytotoxicity (Figure 4(C)). Figure 4(D) illustrates the pathways that POU2F2 may participate in, as derived from GSEA on gene sets. Pan-cancer interrogation of metabolic pathways revealed that elevated POU2F2 transcription robustly co-segregates with IL-6/JAK/STAT3 signaling cascades across multiple independent cohorts (Figure 4(E)). Finally, we evaluated the correlation between POU2F2 expression and fourteen canonical oncogenic hallmarks, revealing the strongest positive association with the inflammation-related signature (Figure 4(F,G)).

### Analysis from pan-cancer to KIRC reveals the role of POU2F2 in tumor immune regulation

**Figure 5.**
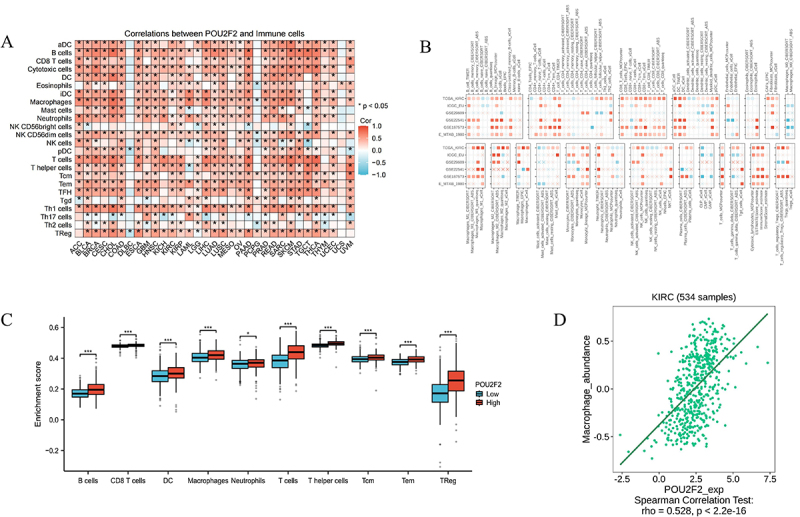
Correlation between POU2F2 expression and immune infiltration in KIRC. (A) Correlation of POU2F2 with immune cells in various cancers. (B) Correlations between POU2F2 and immune infiltrates in KIRC across diverse databases. (C) the association between POU2F2 expression and 10 types of immune cells in KIRC. (D) the expression level of POU2F2 was significantly positively correlated with macrophages. (*p < .05, **p < .01, and ***p < .001).

To elucidate the immunomodulatory function of POU2F2, we first interrogated pan-cancer associations between POU2F2 transcript abundance and the relative densities of infiltrating immune subpopulations. This comprehensive survey revealed that the immune subsets whose infiltration patterns most tightly covaried with POU2F2 levels were strikingly tumor-type–specific (Figure 5(A)). In KIRC, POU2F2 exhibited pronounced positive correlations with most immune cell populations, including B cells, T cells, and macrophages (Figure 5(B)). Figure 5(C) illustrates that, when tumors were stratified into high- versus low-POU2F2-expressing cohorts, the enrichment scores of 10 distinct immune cell subsets diverged markedly, all exhibiting statistically significant disparities. Figure 5(D) reveals a robust positive correlation between POU2F2 expression and macrophage infiltration.

### ScRNA-seq and spatial transcriptomics analysis of POU2F2 in KIRC

**Figure 6.**
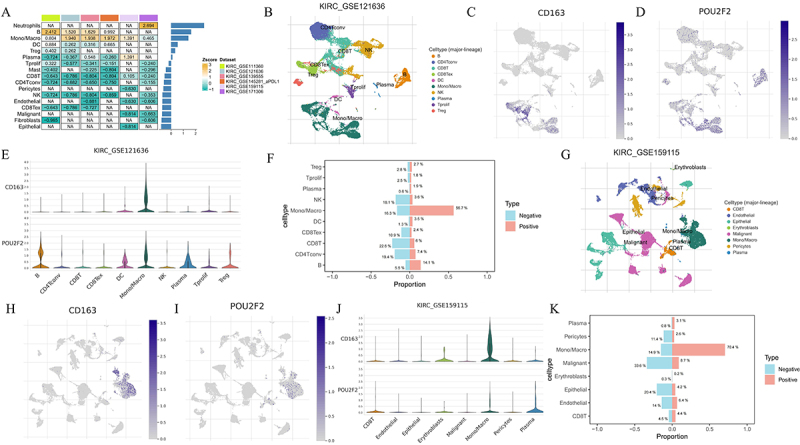
Expression profile of POU2F2 at the single cell level. (A) Pan-cancer single-cell expression of POU2F2. (B–F) Cellular composition and expression of POU2F2 and CD163 in GSE121636 along with proportional distributions in POU2F2-positive versus POU2F2-negative subsets. (G–K) Cellular composition and expression of POU2F2 and CD163 in GSE159115 along with proportional distributions in POU2F2-positive versus POU2F2-negative subsets.

**Figure 7.**
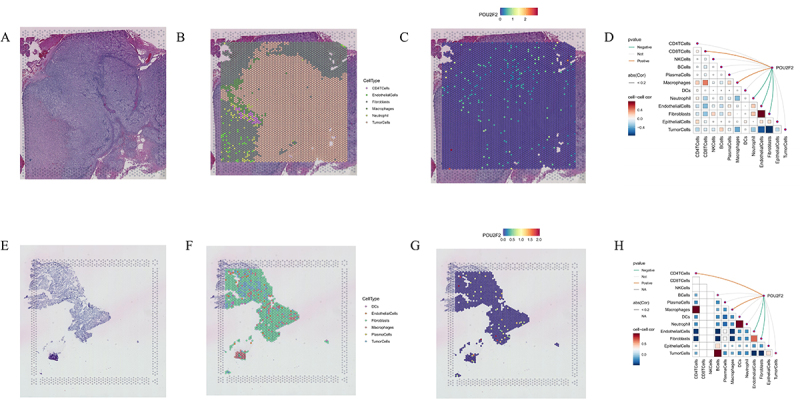
Expression profile of POU2F2 based on spatial transcriptomic analysis. (A–D) the distribution and expression patterns of POU2F2 in KIRC patient-8 of GSE175540. (E–H) the distribution and expression patterns of POU2F2 in KIRC patient-25 of GSE175540.

Leveraging the TISCH database, we delineated the single-cell expression profile of POU2F2, and Figure 6(A) presents a comprehensive single-cell landscape of POU2F2 expression within KIRC. Analyses of GSE121636 and GSE159115 revealed that POU2F2 is predominantly expressed in CD163^+^ macrophages, with a significantly higher macrophage fraction in the POU2F2-positive cohort compared with the POU2F2-negative group (Figures 6(B–K)). Leveraging the spatial transcriptomic dataset GSE171351, we mapped the spatial distribution and expression pattern of POU2F2 in two renal carcinoma patients. The data revealed that POU2F2 is predominantly expressed by macrophages, and its abundance exhibits a robust positive correlation with local tumor cell density (Figures 7(A–H)).

### Knockdown of POU2F2 inhibits malignant biological behavior of KIRC cells

**Figure 8.**
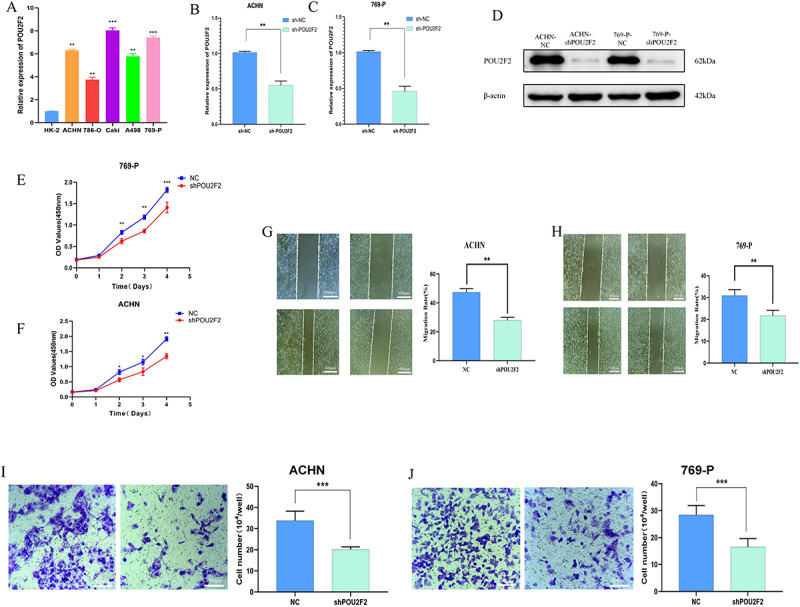
POU2F2 depletion markedly suppressed the proliferative and migratory capacities of KIRC cells. (A) the mRNA expression levels of POU2F2 in KIRC cell lines. (B–D) POU2F2 knockdown in ACHN and 769-P cells was validated by qRT-PCR and Western blot. (E, F) Cell proliferation was assessed by the CCK-8 assay at 24, 48, 72, and 96 h after POU2F2 knockdown in KIRC cells. (G, H) the migratory capacity of KIRC cells after POU2F2 knockdown was evaluated using a wound-healing assay. (I, J) the invasive capacity of KIRC cells following POU2F2 knockdown was assessed using a Transwell invasion assay. *p < .05; **p < .01; ***p < .001.

To elucidate the biological functions of POU2F2, we first validated its expression in renal cancer cell lines. Compared with HK-2 cells, POU2F2 mRNA levels were significantly upregulated across multiple cell lines (Figure 8(A)). ACHN and 769-P cells were selected for POU2F2 knockdown using targeted shRNAs, and qRT–PCR confirmed efficient silencing, with a marked reduction in mRNA expression (Figure 8(B,C)). We have performed Western blot analysis to verify the decreased POU2F2 protein expression after shRNA-mediated knockdown in both ACHN and 769-P cell lines (Figure 8(D) and Supplementary Figure S1). CCK-8 assays showed that POU2F2 depletion significantly suppressed the proliferative capacity of ACHN and 769-P cells relative to controls (Figure 8(E,F)). Reduced POU2F2 expression also significantly decreased the migratory ability of ACHN and 769-P cells (Figure 8(G,H)). Moreover, Transwell assays demonstrated that inhibition of POU2F2 markedly attenuated the invasive potential of KIRC cells (Figure 8(I,J)). Collectively, these findings indicate that POU2F2 knockdown substantially restrains malignant phenotypes in KIRC cells.

### Knockdown of POU2F2 in KIRC cells co-cultured with macrophages inhibits macrophage polarization to M2 type

**Figure 9.**
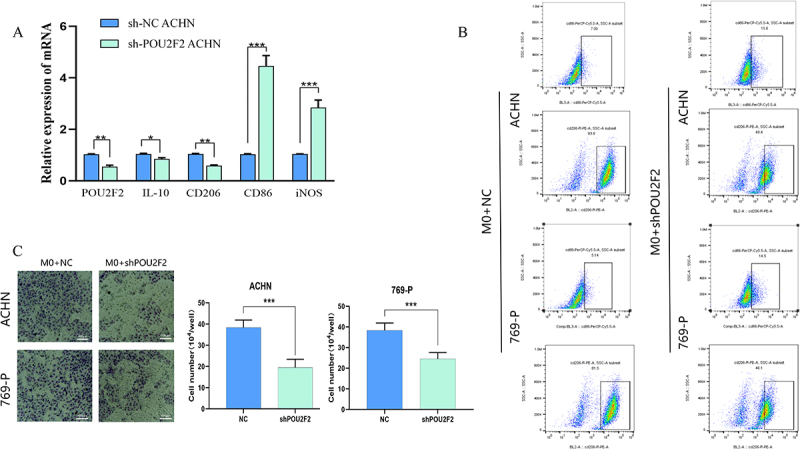
POU2F2 suppression in KIRC cells attenuated macrophage migration and impeded polarization toward an M2 phenotype. (A) in POU2F2-silenced macrophages, the expression of M2-associated genes was decreased, whereas that of M1-associated genes was increased. (B) Macrophages were cultured with conditioned media from KIRC cells with or without POU2F2 knockdown, and CD86 and CD206 expression was quantified by flow cytometry. (C) Macrophages were co-cultured with KIRC cells with or without POU2F2 knockdown in a Transwell system, and macrophage migration was assessed after 24 h. *p < .05; **p < .01; ***p < .001.

We investigated the impact of POU2F2 on macrophage polarization. To this end, THP-1–derived M0 macrophages were cultured with conditioned media collected from ACHN and 769-P cells. The expression of macrophage surface markers was assessed across groups by qRT–PCR and flow cytometry (Figure 9(A)). The changes in cytokine levels in KIRC cells with POU2F2 gene silencing in the co-culture system are shown in Supplementary Figure S2. Co-culture with conditioned media from POU2F2-silenced KIRC cells markedly increased the M1 marker CD86 while significantly decreasing the M2 marker CD206 (Figure 9(B)). In addition, macrophage migratory capacity was evaluated using a Transwell co-culture system, with macrophages seeded in the upper chamber and POU2F2-knockdown or control KIRC cells in the lower chamber. Macrophages exhibited significantly reduced migration toward POU2F2-silenced KIRC cells (Figure 9(C)). Collectively, POU2F2 knockdown in KIRC cells suppresses macrophage migration and inhibits macrophage polarization toward an M2 phenotype.

## Discussion

KIRC remains a formidable clinical challenge due to its high metastatic potential, metabolic heterogeneity, and resistance to conventional chemoradiotherapy. While immune checkpoint blockade has revolutionized the treatment landscape, a significant subset of patients exhibits primary or acquired resistance, often attributed to a highly immunosuppressive TME. In this study, we integrated multi-omics profiling, spatial transcriptomics, and in vitro functional validation to systematically elucidate the oncogenic and immunomodulatory roles of the POU-domain transcription factor POU2F2 in KIRC. Our findings establish POU2F2 not merely as a prognostic biomarker but as a multifaceted driver of tumor progression. We demonstrated that POU2F2 is significantly upregulated in KIRC tissues and correlates with advanced clinicopathologic features and poor survival outcomes. Mechanistically, POU2F2 promotes malignant behaviors–including proliferation, migration, and invasion–within renal cancer cells. Crucially, we unveiled a novel paracrine axis wherein POU2F2 expression in tumor cells orchestrates the polarization of TAMs toward an immunosuppressive M2 phenotype, likely via the modulation of cytokine signaling pathways such as JAK-STAT. These results position POU2F2 as a critical nexus regulating the crosstalk between malignant nephrons and the immune stroma.

The upregulation of POU2F2 in KIRC compared to normal tissue, confirmed here across TCGA and GEO cohorts, aligns with its emerging role as a context-dependent oncogene. While POU2F2 is canonically recognized for its indispensability in B-cell lineage specificality and immunoglobulin gene regulation, its aberrant expression in non-lymphoid epithelial malignancies suggests a ‘co-option’ of developmental transcription factors to fuel tumorigenesis [6,14]. Our data revealed that elevated POU2F2 levels parallel stepwise increases in tumor stage, grade, and metastatic burden. This association was robust enough to anchor a predictive nomogram with high calibration accuracy, suggesting that POU2F2 quantification could refine risk stratification in clinical practice, particularly for patients with indeterminate prognosis based on TNM staging alone.

At the cellular level, our function experiments in ACHN and 769-P cells provided direct evidence of POU2F2’s contribution to tumor aggressiveness. The significant reduction in cell viability, migration, and invasion following POU2F2 silencing mirrors findings in glioblastoma, where POU2F2 promotes survival via the PI3K/AKT/mTOR axis, and contrasts with NSCLC, where it can curb tumor growth by enhancing T-cell infiltration [5,15]. This dichotomy underscores the tissue-specific nature of POU-domain factors. In the context of KIRC, often characterized by VHL loss and HIF-2α accumulation, the ability of POU2F2 to drive invasion suggests it may cooperate with EMT-inducing transcription factors [16,17]. Although we did not explicitly test the ZEB1/2 axis in this study, previous literature indicates that POU2F2 can bind enhancer regions of EMT effectors [4]. Our observation that POU2F2 knockdown impairs the invasive capacity of KIRC cells implies that POU2F2 may be required for the maintenance of the mesenchymal state, potentially facilitating the initial steps of the metastatic cascade [18].

Perhaps the most consequential finding of our study is the role of POU2F2 in remodeling the immune microenvironment. KIRC is one of the most immune-infiltrated solid tumors, yet it paradoxically thrives by fostering an immunosuppressive niche dominated by myeloid cells [19,20]. Our pan-cancer analysis initially flagged a strong correlation between POU2F2 and macrophage abundance. Further resolution provided by single-cell RNA sequencing and spatial transcriptomics from the TISCH and GSE datasets validated this link, demonstrating a physical and functional proximity between POU2F2-expressing cells and CD163+ macrophages.

Our co-culture experiments offer a mechanistic explanation for this association. We observed that conditioned media from POU2F2-intact KIRC cells effectively polarized THP-1 macrophages toward a CD206+ M2-like phenotype, whereas media from POU2F2-knockdown cells favored a CD86+ M1-like shift. M2 macrophages are well-documented culprits in KIRC progression; they secrete VEGF to support angiogenesis, release metalloproteinases to remodel the matrix for invasion, and express PD-L1/L2 to exhaust cytotoxic T cells [7,21]. Although the precise soluble mediators remain to be fully characterized, our pathway enrichment analyses point strongly toward the JAK-STAT signaling axis and cytokine-cytokine receptor interactions. It is plausible that POU2F2 functions as a transcriptional activator for cytokines such as IL-6, IL-10, or M-CSF (CSF-1), which are canonical drivers of M2 differentiation [22]. The strong correlation with the inflammation signature observed in our broad-spectrum analysis further supports the hypothesis that POU2F2 is a regulator of cancer-associated chronic inflammation, bridging intrinsic oncogenic signaling with extrinsic immune editing [23].

The duality observed in the scRNA-seq data—where POU2F2 is expressed both in tumor cells and intrinsic macrophage populations—adds a layer of complexity. While our in vitro focus was on tumor-cell-driven education of macrophages, the expression of POU2F2 within TAMs themselves suggests an autocrine loop might also be at play [24]. In lymphoid cells, POU2F2 regulates differentiation; in TAMs, it may be repurposed to maintain the immunosuppressive transcriptional program, preventing repolarization to an anti-tumor state [25]. This potential ‘double-hit’—where POU2F2 drives tumor invasiveness while simultaneously locking macrophages in a pro-repair, tolerance-inducing state—makes it an exceptionally attractive therapeutic target [26].

The KEGG and GSEA analyses consistently highlighted the enrichment of the JAK-STAT signaling pathway and metabolic reprogramming networks in POU2F2-high tumors. The JAK-STAT pathway is a central communication node in KIRC, often constitutively activated to support survival and immune evasion [27]. POU2F2’s association with this pathway suggests a feed-forward loop. For instance, STAT3 is known to directly bind the POU2F2 promoter in other contexts, and reciprocally, POU2F2 may sustain STAT3 phosphorylation by regulating upstream cytokine production. The ‘Warburg effect,’ characterized by aerobic glycolysis, is prevalent in KIRC due to VHL loss. Interestingly, POU2F2 has been linked to glycolytic regulation in GBM. Our metabolic gene set analysis confirms that POU2F2 correlates with metabolic perturbations in KIRC [28]. It is tempting to speculate that POU2F2-driven metabolic byproducts (e.g., lactate) from tumor cells might further enforce the M2 polarization of neighboring macrophages, creating a metabolic symbiosis that shields the tumor from immune surveillance.

Recent spatially resolved analyses have further highlighted the critical role of macrophage-mediated immune tolerance in ccRCC. Ge et al. reported that spatially segregated APOE+ macrophages foster an immunosuppressive tumor microenvironment and contribute to immune checkpoint blockade resistance through TGF-β signaling and T-cell exhaustion. These findings are highly complementary to our results, which suggest that POU2F2-expressing KIRC cells promote an M2-like macrophage phenotype through a paracrine mechanism, potentially involving JAK–STAT-dependent cytokine signaling. Together, these observations support the concept that distinct but convergent TAM-associated programs, including the APOE+ macrophage axis and the POU2F2-driven M2-polarization loop, cooperate to establish immune escape in ccRCC. Targeting these macrophage-centered immunosuppressive circuits may therefore provide new opportunities to overcome resistance to immunotherapy [29]. Meng et al. proposed a Multi-omic Subtype classification that distinguishes immune cold, hot, and exhausted ccRCC subtypes, thereby providing a useful framework for interpreting tumor immune states and potential responses to immune checkpoint blockade [30]. In this context, our POU2F2-based stratification may offer complementary information. POU2F2-high tumors showed enrichment of immune- and inflammation-related programs, including cytokine–cytokine receptor interaction, JAK–STAT signaling, T-cell receptor signaling, natural killer cell-mediated cytotoxicity, IL-6/JAK/STAT3 signaling, and inflammation-associated signatures. These features suggest that the POU2F2-high subgroup is unlikely to simply reflect an immune-cold phenotype. Instead, given its association with immune infiltration together with immunosuppressive myeloid/TAM-related patterns, POU2F2-high KIRC may partially resemble an inflamed but functionally exhausted immune subtype. Therefore, POU2F2 may serve as a candidate marker for identifying ccRCC tumors with immune activation accompanied by immune dysfunction, a state that may be particularly relevant for ICI patient selection or for designing combinatorial strategies targeting both immune checkpoints and macrophage-centered immunosuppressive circuits.

Furthermore, the PPI network identified interactions with DNA repair proteins (XRCC6) and nuclear receptors (ESR1). This suggests POU2F2’s influence extends beyond transcriptional initiation to chromatin remodeling and genomic stability. If POU2F2 aids in repairing replication stress induced by rapid proliferation, its inhibition could induce synthetic lethality in combination with PARP inhibitors or DNA-damaging agents, an avenue worth exploring in future KIRC trials.

The identification of POU2F2 as a driver of M2 macrophage polarization has profound implications for immunotherapy. Current standard-of-care regimens for metastatic KIRC often combine Tyrosine Kinase Inhibitors (TKIs) with Immune Checkpoint Inhibitors (ICIs) (e.g., Axitinib + Pembrolizumab) [31]. However, dense infiltration of M2 macrophages is a known mechanism of resistance to PD-1 blockade, as these cells can sequester monoclonal antibodies or inhibit T-cell effector function via arginase production [32,33]. Yin et al. reported that HOOK1 suppresses renal cell carcinoma progression through the TGF-β and TNFSF13B/VEGF-A axis and enhances sensitivity to sunitinib and nivolumab, supporting the feasibility of targeting molecular programs that connect tumor progression, angiogenic signaling, and immune regulation [34]. By targeting POU2F2, it may be possible to reprogram the tumor microenvironment by shifting macrophage polarization from M2 to M1, thereby restoring antigen presentation and creating a ‘hot’ tumor milieu that is more responsive to immune checkpoint inhibitors; simultaneously, it could inhibit metastasis by directly reducing the invasive capacity of cancer stem-cell-like populations that often express POU factors, and enhance prognostication by applying the developed nomogram to identify high-risk patients who may benefit from more aggressive adjuvant therapies after nephrectomy.

This study benefits from a robust design integrating large-scale bioinformatic mining with extensive experimental validation. The use of spatial transcriptomics allows for a direct visualization of the tumor-immune interface, overcoming the spatial loss inherent in bulk sequencing. Furthermore, the construction and validation of a prognostic nomogram provide an immediate translational tool for clinicians. However, several limitations must be acknowledged. First, although we validated the macrophage repolarization effect in vitro, the complex milieu of the TME—including T cells, NK cells, and fibroblasts—cannot be fully replicated in a 2D co-culture system. Although we observed that POU2F2 knockdown inhibits migratory and invasive capacities—phenotypes often associated with EMT—we did not directly evaluate canonical epithelial and mesenchymal markers. Future studies incorporating the analysis of these markers will be necessary to confirm the role of POU2F2 in EMT regulation. The specific cytokine(s) responsible for the paracrine effect remain to be isolated via antibody neutralization assays or proteomic secretome analysis. Second, while our findings rely on human cell lines, validation in syngeneic mouse models with intact immune systems is requisite to confirm whether POU2F2 inhibition sensitizes tumors to immunotherapy. Finally, the retrospective nature of the TCGA and GEO cohorts, while powerful, warrants confirmation in prospective clinical cohorts to validate the prognostic utility of POU2F2 protein staining in varied clinical settings.

## Conclusion

This study identifies POU2F2 as a key oncogenic driver in kidney renal clear cell carcinoma (KIRC) that links tumor aggressiveness with immune evasion. Elevated POU2F2 predicts poor prognosis and promotes proliferation and invasion. Mechanistically, POU2F2-expressing tumor cells foster an immunosuppressive microenvironment by skewing macrophages toward an M2-like phenotype, likely through JAK–STAT-dependent cytokine signaling. Targeting POU2F2 and its downstream pathway may help overcome immunotherapy resistance, warranting development of POU2F2 inhibitors and evaluation of combination strategies.

## Supplementary Material

Supplemental Material

tableS1.doc

## Data Availability

The datasets used and/or analyzed during the current study are available from the corresponding author on reasonable request.
