# Supplementary material for: POU2F2 as a dual oncogenic and immunomodulatory driver in kidney renal clear cell carcinoma
Source: Cell Adh Migr. 2026 Jul 8;20(1):2700062. doi: 10.1080/19336918.2026.2700062 (PMC13353782; doi:10.1080/19336918.2026.2700062)
Supplement: tableS1.doc [file KCAM_A_2700062_SM0441.doc]

| Table S1. shRNA target sequences against human POU2F2. | | | |
| --- | --- | --- | --- |
| Marker | Gene | Gene ID | TargetSeq |
| sh1 | POU2F2 | 5452 | GCTACCGACACCAAATCTATT |
| sh2 | POU2F2 | 5452 | GAAATGGACCAGACACTAATC |
| sh3 | POU2F2 | 5452 | ACTTCAGCCAGACGACCATTT |
| NC | NC | NA | CCTAAGGTTAAGTCGCCCTCG |
